# Performance of International Medical Students In psychosocial medicine

**DOI:** 10.1186/s12909-017-0950-z

**Published:** 2017-07-10

**Authors:** D. Huhn, J. Lauter, D. Roesch Ely, E. Koch, A. Möltner, W. Herzog, F. Resch, S. C. Herpertz, C. Nikendei

**Affiliations:** 1 Department of General Internal Medicine and Psychosomatics, Centre for Psychosocial Medicine, University Hospital Heidelberg, Thibautstraße 4, D-69115 Heidelberg, Germany; 2 Department of General Psychiatry, Centre for Psychosocial Medicine, University Hospital Heidelberg, Heidelberg, Germany; 3 Department of Child and Adolescent Psychiatry, Centre for Psychosocial Medicine, University Hospital Heidelberg, Heidelberg, Germany; 4 Competence Centre for Examinations in Medicine, Heidelberg, Baden-Württemberg Germany

**Keywords:** International medical students, OSCE, Examination performance, Psychosocial medicine, Conversational skills

## Abstract

**Background:**

Particularly at the beginning of their studies, international medical students face a number of language-related, social and intercultural challenges. Thus, they perform poorer than their local counterparts in written and oral examinations as well as in Objective Structured Clinical Examinations (OSCEs) in the fields of internal medicine and surgery. It is still unknown how international students perform in an OSCE in the field of psychosocial medicine compared to their local fellow students.

**Methods:**

All students (*N* = 1033) taking the OSCE in the field of psychosocial medicine and an accompanying written examination in their eighth or ninth semester between 2012 and 2015 were included in the analysis. The OSCE consisted of four different stations, in which students had to perform and manage a patient encounter with simulated patients suffering from 1) post-traumatic stress disorder, 2) schizophrenia, 3) borderline personality disorder and 4) either suicidal tendency or dementia. Students were evaluated by trained lecturers using global checklists assessing specific professional domains, namely building a relationship with the patient, conversational skills, anamnesis, as well as psychopathological findings and decision-making.

**Results:**

International medical students scored significantly poorer than their local peers (*p* < .001; η^2^ = .042). Within the specific professional domains assessed, they showed poorer scores, with differences in conversational skills showing the highest effect (*p* < .001; η^2^ = .053). No differences emerged within the multiple-choice examination (*p* = .127).

**Conclusion:**

International students showed poorer results in clinical-practical exams in the field of psychosocial medicine, with conversational skills yielding the poorest scores. However, regarding factual and practical knowledge examined via a multiple-choice test, no differences emerged between international and local students. These findings have decisive implications for relationship building in the doctor-patient relationship.

## Background

According to the OECD (Organisation for Economic Cooperation and Development), the number of international students has increased worldwide, from 0.8 million in 1975 to 4.5 million in 2012. The countries with the highest percentages of international students are Luxembourg, Australia, the United Kingdom, Switzerland, Austria and New Zealand. More than half of these students come from Asia, especially from China, which accounts for the largest number of citizens enrolled abroad [1]. Moreover, international students have come to make up a significant proportion of medical school undergraduates worldwide in the field of medical education [2]. In a comparison of OECD countries, health sciences were found to be the third most attractive field among international students [1]. In Germany, international students account for up to 15% of all medical students, with over 2000 young people beginning their studies each year [3].

It has been documented that international students face several challenges during their studies due to language and cultural barriers [4, 5] in terms of adjusting to their new homes. Regarding their examination performance at university, it has been shown that international medical students achieve lower results in pre-clinical written tests [6], in clinical examinations [7] as well as in state examinations [6]. Moreover, several studies have demonstrated poorer results of international students concerning performance in Objective Structured Clinical Examinations (OSCEs) in the fields of internal medicine and surgery [7–10]. In two British studies, it was shown that students of Asian origin [8] and ethnic minorities in general [10] – both groups being educated in the UK, using English as their first language – performed weaker in an OSCE assessing procedural and communication skills than their white counterparts. Wass and colleagues [10] proposed that these differences were explained, in particular, by poorer communicative styles within the conversations with simulated patients. In an Australian OSCE study with a specific focus on communication skills, both students of non-Western origin and students who did not have English as their mother tongue were found to perform poorer than their Western, English-speaking counterparts – especially in terms of assessed communication skills [7]. Moreover, Mann et al. [9] reported poorer results of international students in first- and second-year OSCEs in an Australian Medical School – even in international students who had identified English as their language family. The authors, therefore, argued that it is possibly not the language acquisition in a foreign country itself that is a major predictor of academic performance, but rather international students’ acculturation [9]. However, to the best of our knowledge, so far, no studies have been conducted on international students’ OSCE performance either for non-English speaking countries or for the field of psychosocial medicine.

In the field of psychosocial medicine, communicative skills represent the core tool, as they are the supporting pillar in history taking, diagnosis and also in counseling and therapy. In a sense, the spoken word can be seen here as therapy itself [11]. Psychosocial medicine covers a wide range of disorders from psychiatric, psychosomatic and psychotherapeutic to somato-psychological and somatoform disorders. Competencies in this field, therefore, also have important implications for the detection of comorbidities in somatically ill patients and, thus, for appropriate treatment, as psychiatric comorbidities weaken the prognosis for the diseases course (e.g. in chronic cardiac insufficiency) [12]. Moreover, every doctor should have the conversational competencies to cope with interactionally difficult patients or to detect endangerment of self and others. Due to the above-mentioned language difficulties, international medical students might be particularly challenged in the field of psychosocial medicine. Therefore, a needs assessment focusing on these aspects seems to be essential. The presented study is the first to report OSCE results of fourth-year international students for the field of psychosocial medicine. Moreover, the study allows a further differentiation regarding aspects, such as relationship building, conversational skills, structured and accurate anamnesis, as well as psychopathological findings and decision-making.

The aim of the current study was to compare the performance of German, EU (European Union) and non-EU medical students in both a psychosocial OSCE and an accompanying written examination in the clinical part of their studies at the Medical Faculty of Heidelberg. We assumed that students both (1) with an EU background and (2) without an EU background would perform poorer than German students in the psychosocial OSCE as well as the written examination, that (3) this performance would be weaker in non-EU than in EU students, and (4) that the weaker OSCE performance of EU and non-EU students would be more pronounced for language-related aspects, like ‘conversational skills’.

## Methods

### Study design

The present study is a retrospective analysis of medical students’ performance in an OSCE in psychosocial medicine as well as an accompanying written examination in the clinical part of their studies at the Medical Faculty of Heidelberg. The OSCE scores and the written examination results for the period 2012–2015 were analysed. The obtained data were combined with information about students’ origin (migration background, differentiated by EU and non-EU states).

### Data collection

All data regarding performance in the OSCE and the written exam were provided by the teaching coordinator of the psychosocial module as well as the Deanery of Student Affairs at the Medical Faculty Heidelberg. Only data of students taking part in the tests for the first time were entered into the analysis; participants repeating a test after failing to pass the first time were not considered.

### Demographics of cultural background within the samples

Two pieces of information were available to determine the students’ origin: their nationality and their place of birth. If one of these two variables was non-German, a further differentiation according to EU or non-EU countries was made. Additionally, the following countries of origin were included in the category “German”: Austria, German-speaking Switzerland, Liechtenstein and Luxembourg. As all of these states are at least partly German-speaking, it can be assumed that these students should not show any great difficulties in adapting. Thus, based on the methodological procedure described above, the following categories were formed: students with (i) a German-speaking origin, (ii) an EU migration background, and (iii) a non-EU migration background. This restricted validity of category choice stemmed from the retrospective nature of the conducted study, in which it was impossible to ask for further details about students’ cultural background.

### Psychosocial medicine OSCE

In line with the OSCE guidelines by Patrício and colleagues [13], the analysed OSCE in psychosocial medicine is part of the mandatory psychosocial module for students in their eighth or ninth semester at the Centre for Psychosocial Medicine, University Hospital Heidelberg. The Centre consists of five different departments: General Psychiatry, General Internal Medicine and Psychosomatics, Child and Adolescent Psychiatry, Institute for Medical Psychology, and Institute for Psychosocial Prevention. The first three of these contributed to the conception of the four-week-long psychosocial module, comprising lectures and seminars with patient presentations, clinical placements in hospitals, classes on problem-oriented learning (POL) [14] and communication training with standardized patients [15]. Each module was attended by 30 to 40 students and culminated in the OSCE as well as the written examination. The OSCE comprised four different stations concerning the following topics:

- post-traumatic stress disorder

schizophrenia

- borderline personality disorder

suicidal tendency or dementia

At each of the four stations, students faced one standardized patient with the respective disorder and were asked to establish contact with the patient and take his or her history. Students had nine minutes at each station, with two minutes between each station to change rooms. One examiner at each station assessed students’ performance using tablet-based global checklists. These checklists addressed the following specific professional domains of students’ performance (the number of possible points is given in brackets):

- relationship with the patient (maximum 5 points): introduces himself (1), friendly and approachable (1), takes patient seriously (1), non-judgemental (1), shows understanding for patient’s situation (1)

conversational skills (maximum 5 points): establishes rapport with the patient and is empathetic (1), is clear and definite (1), encourages patient to answer (1), builds up trust (1), reflects important issues (1)

- anamnesis (maximum 5 points): works in a structured way (1), asks central questions of brief anamnesis (social situation, school career, development of symptoms etc.) (4)

psychopathological findings and resulting decision-making (maximum 10 points): addresses symptoms and impairments openly (1), determines orientation (2) and cognitive impairments (2), determines the extent of the problem/ suicidality (1), decides on further measures (inpatient admission) (2), discusses further steps adequately with the patient (2)

At each station, students could gain a maximum of 25 points, resulting in a sum of 100 points over all four stations. To pass the exam, students had to achieve at least 60 points. After assessing the examination’s outcome, students could seek detailed feedback on their results.

### Psychosocial medicine written examination

The written exam brought the mandatory psychosocial module to a close and consisted of 48 multiple-choice questions (36 with single correct answers, 10 with multiple selections) in three different categories: psychiatry (26 questions), psychosomatics (16 questions) and child and adolescent psychiatry (6 questions). Students had 70 min to complete the questionnaire. They could achieve a maximum of 48 points; each correct answer was awarded one point, and in multiple-selection questions, half points for partially correct answers were also possible. To pass the exam, students had to give at least 60% correct answers.

### Statistical analysis

Data analysis was conducted using the “Statistical Package for the Social Sciences” (SPSS) for Windows, version 22. To compare the different groups regarding the achieved performance in the OSCE or the written examination, ANOVAs (Analyses of Variance) with repeated measures were calculated. To detect differences between the three groups (German vs. EU, German vs. non-EU, EU vs. non-EU), contrast analyses were subsequently undertaken. Cronbach’s alpha was calculated to determine the reliabilities of the OSCE and the written exams.

## Results

### Sample description

The sample comprised 1034 medical students in their eighth or ninth semester at the University of Heidelberg. For the OSCE, one examinee had to be excluded from the study because, for unknown reasons, there were no available data concerning his achieved grades. 840 of the remaining 1033 students were German-speaking. 73 had an EU migration background and 120 had a non-EU migration background. For the accompanying written examination, only the data of 995 of the initial 1033 students were available. 808 of this remaining group had a German background, 72 came from a country within the EU and 115 from countries outside the EU (see Table 1).

**Table 1:** Descriptive presentation of sample

Subject	Cultural background	*N*	Male	Female	*M*_age_	*SD*_age_
OSCE	German	840	413	427	25.33	3.25
	EU	73	22	51	24.65	2.94
	non-EU	120	60	60	25.03	2.81
	all	1033	495	538	25.26	3.19
Written examination	German	808	396	412	25.35	3.27
	EU	72	22	50	24.54	2.87
	non-EU	115	58	57	25.03	2.87
	all	995	476	519	25.27	3.21

### Examination performance differences between German and international students

#### Performance in the OSCE

To calculate the overall performance in the OSCE, ANOVAs were used. The comparison between German students, students with an EU migration background and students with a non-EU migration background showed a significant group effect (*F(2, 1030)* = 23.524: *p* < .001; *η* ^*2*^ = .044). According to contrasts, both non-EU (*p* < .001; *d* = .61) and EU students (*p* < .001; *d* = .44) scored significantly worse than their German colleagues (see Table 2).

**Table 2:** ANOVAs for scores in the OSCE as well as the written examination in German, EU and non-EU students

Subject	Groups	*N*	*M*_score_	*SD*_score_	Root mean square	*F*	*p*	*η* ^*2*^
OSCE, total score	German	840	88.08	5.21	682.765	23.524	.000	.044
	EU	73	85.75	5.83				
	non-EU	120	84.82	6.27				
OSCE, ‘relationship with the patient’	German	840	19.24	1.02	9.298	8.471	.000	.016
	EU	73	19.04	1.10				
	non-EU	120	18.84	1.21				
OSCE, ‘conversational skills’	German	840	18.23	1.49	74.133	28.767	.000	.053
	EU	73	17.44	1.72				
	non-EU	120	17.17	2.19				
OSCE, ‘anamnesis’	German	840	15.94	2.05	20.069	4.699	.009	.009
	EU	73	15.32	2.18				
	non-EU	120	15.55	2.10				
OSCE, ‘psychopathological findings and decision-making’	German	840	34.66	2.91	110.676	12.558	.000	.024
	EU	73	33.97	3.35				
	non-EU	120	33.45	3.11				
written examination	German	808	43.07	2.33	14.246	2.070	.127	.004
	EU	72	42.57	5.24				
	non-EU	115	42.70	2.05				

Regarding the ‘relationship with the patient’ items, groups also differed significantly from each other (*F(2, 1030)* = 8.471: *p* < .001; *η* ^*2*^ = .016), with non-EU students scoring significantly poorer than German students (*p* < .001; *d* = .39). No significant results emerged for students from EU countries (*p* = .106; *d* = .20) (see Table 2).

With respect to ‘conversational skills’, group differences again proved to be significant (*F(2, 1030)* = 28.767: *p* < .001; *η* ^*2*^ = .053). According to contrasts, students from EU countries (*p* < .001; *d* = .52) as well as those from non-EU countries (*p* < .001; *d* = .67) scored significantly poorer than their German counterparts (see Table 2).

In terms of the ‘anamnesis’ performance, again, there was a significant group effect (*F(2, 1030)* = 4.699: *p* < .05; *η* ^*2*^ = .009) and again, students with an EU migration background (*p* < .05; *d* = .31) as well as those with a non-EU background (*p* < .05; *d* = .20) scored significantly poorer than German students (see Table 2).

Scoring in ‘psychopathological findings and decision-making’ also showed a significant group effect (*F(2, 1030)* = 12.558: *p* < .001; *η* ^*2*^ = .024). Students from non-EU countries scored poorer than German students (*p* < .001; *d* = .47). No significant results emerged for EU students (*p* = .054; *d* = .24) (see Table 2).

#### Performance in the written examination

The analysis of variance for the performance in the written examination showed no significant differences between groups (*F(2, 1030)* = 2.070: *p* = .127; *η* ^*2*^ = .004) (see Table 2).

### Reliabilities of OSCE and written examination

According to literature, a reliable OSCE should consist of at least twelve stations [16]. Due to limited resources in the psychosocial OSCE, only four stations could be provided as described above, resulting in a rather low internal consistency with a Cronbach’s alpha of α = .58. Also, the reliability of the written examination was rather low, with a Cronbach’s alpha of α = .47.

## Discussion

To the best of our knowledge, the current study is the first to examine international students’ performance in psychosocial medicine – in an OSCE as well as an accompanying written examination. In accordance with other studies, we found that international students scored significantly poorer in the OSCE than local students. Students with a non-EU background scored poorer than their German counterparts in the OSCE total score, but also in all four specific professional domains of performance. Students from countries within the European Union also scored poorer than German-speaking students concerning the total score, but only scored lower in two professional domains: conversational skills and anamnesis. The largest effect emerged in the context of conversational skills, meaning that the differences between German, EU and non-EU students were highest within this category. Of course, one could argue that all these emerging differences are rather small since international and local students mostly only differ up to one point in the scores. However, if we take a look at the effect sizes (see Table 2 and results section) it becomes clear that even these small differences have to be seen as small to moderate effects. In the multiple-choice written examination, no significant differences emerged between the three groups; therefore, their scoring can be seen as equal.

Our research enabled us to replicate previous findings [7–10] showing that international medical students achieve weaker results than local students in OSCEs. In addition, we were able to demonstrate this quite robust effect for the field of psychosocial medicine for the first time. In contrast to other medical disciplines, psychosocial medicine mainly focuses on communication, as here patients mainly talk about their mental health problems. Our data set also enabled us to introduce a distinction between international students from EU and non-EU countries, which represents a novel approach. As was suggested in previous studies [7, 10], there is reason to believe that linguistic difficulties might be the reason for the documented differences, since international students tend to use poorer communicative styles [10]. Evidence for this can also be seen in the fact that the largest differences between the examined groups of students emerged in the context of conversational skills. In this segment, examiners assess the students’ ability to show a well-planned and structured approach, to talk to their patients in a comprehensible and patient-suitable language, to address patients’ emotions openly and to reflect on crucial points. For all of these aspects, a reasonable mastery of the language seems to be indispensable. Although the international students provided a language certificate at the beginning of their studies and, at the time of our study, had already spent at least four years in Germany practising dealing with the foreign language, they still did not fare as well as their local counterparts in a complex task of a free conversation with a mentally impaired patient. The fact that non-EU students performed slightly poorer than students with an EU background might further suggest that communicative differences are at play. It has been documented that students whose mother tongue is closer to the geolinguistic origins of medical terminology (mostly Latin and Greek) show fewer difficulties when dealing with language-based tasks [17]. For example, students who learned Latin at school perform better than students who did not. However, as Long and colleagues [17] evaluated students’ scientific comprehension, these results are hardly comparable with our study; the influence of students’ mother tongue as well as their technical language competence has been shown to be much smaller in regards to the ability to interact with patients in a comprehensible way.

Furthermore, the assumption of a different acculturation process [9] should be discussed. It can be expected that for international students, the larger the cultural differences between home country and chosen centre of life, the greater the initial psychosocial disorientation [18], leading to lower academic grades [19]. However, studies which attribute international students’ poorer results to acculturation processes mostly focus on first- or second-year students [9, 19], while in the current study, students had already spent more than four years in a foreign country and culture. Nevertheless, it is possible that the medical students have not fully acculturated to Germany and, therefore, perform weaker than local students. Another possible interpretation could be that examiner bias or even discrimination may exist [20], with examiners focusing more intensively on the poorer communication styles of international students [10]. This would apply to the present study, particularly since performance differences were highest in language-related domains, like students’ conversational skills, and lowest in less language-related domains, like anamnesis or relationship building.

An additional aspect that might explain the international and German students’ divergent performance could lie in the students’ heterogeneous previous schooling in the different educational systems before coming to Germany. Certainly, significant differences in the quality of the educational systems across the analysed countries can be found. However, we were unable to control for this aspect in this study.

The finding that no significant differences emerged between international and German-speaking students in the written examination speaks for itself and confirms that the differences found in the OSCE are most probably not due to poorer cognitive abilities in international students. Moreover, it must be acknowledged that international students do improve over time, as studies focusing on the preclinical study semesters report significantly poorer performance at this earlier stage [6].

While international students in higher semesters perform more or less equally as well as local students in written examinations, they are still less successful concerning conversations with patients. These findings have important implications for the doctor-patient relationship. Conversation training with simulated patients, in which international students can practice their communicative abilities, might provide a good opportunity to better prepare them for patient encounters.

### Limitations

Limitations of the current study lie in the fact that it was only possible to detect students’ migration background from the parameters “nationality” and “place of birth”. This approach may have led to incorrect assessments concerning the actual migration background. Other studies regarding OSCE performance [7] managed to differentiate their students into Western/non-Western, English/non-English native speakers as well as local/international, since these studies were of a prospective nature. However, case numbers in such studies are rather low. In the course of our retrospective study, we were able generate a larger number of cases, but nevertheless need to take into account this uncertainty. For further prospective studies investigating students’ examination performance, a more differentiated breakdown of international students’ origins would be preferable. Another limiting aspect of the study lies in the fact that the examined OSCE only consists of four stations. With only four different scenarios task, specificity plays an important role. Therefore, the inclusion of further conditions, via more different stations, may result in students performing differently. Further limitations lie in the fact that the results were not controlled for examiner bias. Theoretically, examiners may have rated students from different origins differently. Another limiting aspect is that the study is a retrospective, exploratory analysis which can only support speculations about causal links and backgrounds of the results in terms of a “clarification study” [21]. Thus, there is a need for further research investigating reasons behind the given performance differences. Moreover, it must be taken into account that reliabilities of the OSCE as well as the written examination only show a poor value.

## Conclusion

To our best knowledge, the current study is the first to examine OSCE results of fourth-year international medical students for the field of psychosocial medicine. Moreover, our results allow a further differentiation in several aspects of performance. International students scored significantly poorer than their local counterparts with differences in conversational skills showing the highest effects. In the accompanying multiple-choice examination, no differences emerged between international and local students’ scores. Hence, our findings suggest that international students largely overcome language problems and cultural barriers within their first four years of studying abroad. However, they are still less successful concerning conversations with patients which has important implications for the doctor-patient relationship. Conversation trainings with simulated patients might help to counteract these deficits.

## Abbreviations

ANOVA: Analysis of Variance
e.g.: exempli gratia, meaning ‘for example’
EU: European Union
OECD: Organisation for Economic Cooperation and Development
OSCE: Objective Structured Clinical Examination
POL: Problem-Oriented Learning
UK: United Kingdom
